# Exploring Anhedonia in Kennelled Dogs: Could Coping Styles Affect Hedonic Preferences for Sweet and Umami Flavours?

**DOI:** 10.3390/ani10112087

**Published:** 2020-11-11

**Authors:** Daniela Luna, Carolina Carrasco, Daniela Álvarez, Catalina González, Juan Ignacio Egaña, Jaime Figueroa

**Affiliations:** 1 Departamento de Ciencias Animales, Facultad de Agronomía e Ingeniería Forestal, Pontificia Universidad Católica de Chile, Macul, Santiago 7820436, Chile; dlunavet@gmail.com; 2 Departamento de Fomento de la Producción Animal, Facultad de Ciencias Veterinarias y Pecuarias, Universidad de Chile, Santa Rosa 11735, La Pintana, Santiago 8820000, Chile; carola.acl@gmail.com (C.C.); dalvarezr@ug.uchile.cl (D.Á.); cata2403@gmail.com (C.G.); jegana@uchile.cl (J.I.E.)

**Keywords:** anhedonia, chronic stress, coping style, domestic dogs, monosodium glutamate, sucrose

## Abstract

**Simple Summary:**

Kennelled dogs are susceptible to suffer chronic stress when social interactions with conspecifics and spatial needs are long-term restricted. Chronic stress may affect pleasure perception of food and solutions in dogs as observed in several animals, a phenomenon known as anhedonia. However, little information exists on how different coping styles could prevent the onset of anhedonia. Fourteen kennelled Beagle dogs were used to study the acceptability and preference for different dilute palatable sucrose and monosodium glutamate (MSG) solutions. Coping style of animals was previously evaluated through a modified human-approach test. This test consisted in assessing whether or not dogs approached an unfamiliar human when a feeding opportunity was presented, classifying them as close dogs (CD; proactive) or distant dogs (DD; reactive) respectively. It was observed that DD presented a lower intake of both sucrose and MSG dilute solutions compared with CD. Moreover, DD exhibited a higher consumption of MSG than CD at the highest concentrations, supporting that their intake depends on solution palatability. Finally, DD did not prefer sucrose or MSG solutions over water at any dilute solution offered. Together, these results suggest that dogs that are categorized as reactive animals could diminish their ability to perceive dilute palatable solutions reflecting depressive-like behaviours such as anhedonia.

**Abstract:**

Kennelled dogs are at risk of suffering chronic stress due to long-term spatial, social and feeding restrictions. Chronic stress may decrease the dogs’ capacity to feel pleasure when facing hedonic experiences, modifying their perception for palatable ingredients. However, different abilities to cope with environmental stressors could prevent the onset of anhedonia. Fourteen kennelled Beagle dogs were used to study the acceptability and preference for different dilute sucrose and monosodium glutamate (MSG) solutions. Coping style of animals was previously evaluated through a human approach test (HAT) and classified as close dogs (CD; proactive) or distant dogs (DD; reactive) according to whether or not they approached an unfamiliar human when a feeding opportunity was presented. Consumption results were analysed taking into account the sucrose/MSG concentrations, HAT (CD or DD), age, and weight of the animals. DD presented a lower intake of sucrose (*p* = 0.041) and MSG (*p* = 0.069) solutions compared with CD. However, DD exhibited a higher consumption of MSG than CD at its highest concentrations, supporting that their intake depends on solution palatability. Finally, DD did not prefer sucrose or MSG solutions over water at any dilute solution offered. Together, these results suggest that dogs that are categorized as reactive animals could diminish their ability to perceive dilute palatable solutions, reflecting depressive-like behaviours as anhedonia.

## 1. Introduction

Biologically relevant behaviours are reinforced by pleasant perception, allowing animals to repeat those behaviours in the future [1,2]. Taste sense allows one to recognize foods that give nutrient rewards. However, hedonism experienced during the consumption of a food is not only affected by its sensory characteristics but also by physiological and psychological aspects proper of the individual [3,4]. In humans, it has been described that some pathologies such as schizophrenia and depression decrease the ability to experience pleasure in front of usually pleasant activities, a phenomenon known as anhedonia [5,6]. Anhedonia has also been observed in other non-human animals under chronic stress, where a decrease in the acceptability and/or preference for sucrose is described [5,6,7,8]. In studies carried out in rats, the preference and acceptability for sucrose solutions have been measured before and after being exposed to mild, continuous and rotating stressors, demonstrating a decrease in consumption and preferences of dilute sucrose solutions relative to water [5,9,10]. In other species such as horses, a lower consumption of sugar blocks has been observed in “depressive-like” animals [11]. Moreover, pigs were not capable of preferring dilute sucrose solutions (0.5%) over water, after being mixed with unfamiliar animals or subjected to movement restriction [8]. However, there is little information related to anhedonia in companion animals, despite evidence suggesting that dogs and cats can show signs of “depressive-like behaviours” due to chronic stressors [12,13], which could alter their pleasure perception, reflecting changes in their feeding behaviour.

Dog’s welfare is a current societal concern, attracting increasing attention on how animals are raised and used [14,15]. Companion dogs may present diverse welfare issues, such as spatial and social limitation, lack or insufficient amount of exercise, absence of environmental enrichment, and food restriction, among others [16,17,18,19]. When stressors are maintained across time, dogs may suffer chronic stress affecting behaviours such as feeding [17,20]. This situation could be worse in kennelled dogs, particularly in those housed for longer periods of time, where they usually live under suboptimal conditions and their environment is often restricted in space and complexity and can limit the socialisation with other conspecifics and humans [21,22]. Previous studies have shown through physiological and behavioural measures that dogs experience acute stress after admission to kennels, and some suffer chronic stress when kennelled for long periods [17,23]. This in turn could alter their preferences for palatable foods.

It is known that the ability to cope with stressful and challenging situations is related to welfare in domestic animals [24]. Coping style has been defined as a correlated set of behavioural and physiological responses to challenge that is stable and consistent over time and across situations [25,26]. In a growing number of species, two types of stress response patterns or coping styles have been found [26,27,28]. The proactive response is characterized behaviourally by individuals showing territorial control, high levels of aggression, shorter latencies to explore novel environments or stimuli, and active attempts to counteract stressors [25,29,30]. Additionally, these individuals are more prone to take risks and develop routines [31]. Physiologically, proactive individuals respond with a higher activation of the sympathetic-adrenomedullary system and an increase in noradrenergic stimulation [28]. On the other hand, a reactive response is characterized by immobility, low levels of aggression, longer attack latencies, and an easy adaptation to a changing environment [24,32,33]. Reactive individuals respond with a higher activation of the hypothalamic–pituitary–adrenocortical axis and higher parasympathetic reactivity, increasing circulating glucocorticoids [28,34]. It has been proposed that these characteristics predispose proactive and reactive animals to develop stereotypic and depressive-like behaviours respectively, as a strategy to cope with chronic stress [35,36]. In a study carried out by Zheng and Zheng [37], rats that were classified as reactive when confronted with an acute stressor, suffered from anhedonia when faced with chronic stress. Both strategies serve to dearouse the animal and minimize the effects of stress, but while the appetitive loop in proactive animals will continue until the individual is aroused enough to redirect to stereotypy, in reactive individuals depression will act negatively on motivation and appetitive behaviours, therefore showing anhedonia [36].

We hypothesized that chronically restrained dogs in space, feeding frequency, and social interactions would exhibit a differentiated hedonic perception according to their coping styles. Specifically, reactive dogs at risk of suffering chronic stress under a kennel environment would be more prone to experience anhedonia. On the other hand, the long domestication process in dogs has led to changes in their genetics presenting hedonic reactions and an adequate digestibility in front of sweet carbohydrates such as sucrose [38]. However, its carnivorous origin causes that umami flavours, related to protein compounds, could also generate pleasure reactions at the time of consumption [39]. Due to its high palatability, umami, just like sweet solutions, could be an effective tool to evaluate the possible presentation of anhedonia in these animals such as occurs in rats [7]. The objective of this study was to evaluate if kennelled dogs used in pets’ food industry may modify their preferences for hedonic solutions (sweet or umami) according to their coping style, assessing the latter through a modified human-approach test with a feeding opportunity.

## 2. Materials and Methods

The dogs used in the present experiments belonged to the Research Centre of Pet Feeding Behaviour at the Facultad de Ciencias Veterinarias y Pecuarias (FAVET), Universidad de Chile, located in the Metropolitan region of Chile (Santiago city, 34°21′ S, 71°18′ W). Experimental procedures were approved by the bioethical committee of “Facultad de Ciencias Veterinarias y Pecuarias”, Universidad de Chile (Nº042013).

### 2.1. Animals and Housing

A total of 14, unneutered male (11) and female (3) kennelled Beagle dogs (*Canis lupus familiaris*) were used in two consecutive tests where the acceptability and preference for dilute sucrose and monosodium glutamate (MSG) solutions were studied. Dogs were aged 3–12 years (average 5.2 ± 3.2 years old). A six months training period was performed to all animals at their arrival to the experimental kennel (six month-old) to habituate them to experimental procedures of food preference tests. Dogs were individually housed and fed once a day (09:30 h) a commercial extruded food according to their body condition. Dogs had constant access to fresh water and were exercised daily through 60 min walks by veterinary students. Health status of dogs was assessed prior to the start of the experiments by veterinarians of the Clinical Sciences Department of FAVET and only clinically healthy dogs participated in experimental trials. For more details about general housing, training, feeding, and sanitary conditions of kennelled dogs see Alegría-Morán et al. (2019) [40].

### 2.2. Experimental Procedures

#### 2.2.1. Determination of Coping Style: Human-Approach Test with a Feeding Opportunity

Before conducting acceptability and preference tests, dogs were assessed through a modified human-approach test (HAT) that incorporated a feeding opportunity for the animals. The human-approach test is one of the common measurements used to determine the coping style of animals [41,42,43,44], including domestic dogs [45,46]. In this test, a lower latency to approach the human would indicate in most cases that animals are predominantly proactive. By contrast, animals that exhibit higher latencies are commonly associated with reactive styles and differ from the proactive ones because they are less active and react approaching humans with passivity and submission [46,47].

In our study, HAT with a feeding opportunity was carried out in order to classify dogs according to their coping style (proactive or reactive) based on their approach response to a food source with an unfamiliar person nearby. The experimenter (unknown woman, 1.65 m), who wore a green overall and white boots, quietly entered the room with a plate with pelletized food, leaving it in the front and centre of the kennel floor. Afterwards, the experimenter left the room without turning his back to the animal, closing the door from the outside and remained standing close to it during the next three min. The dogs that remained distant from the plate during this period were assigned to the “distant” dogs (DD) group, whereas the animals that approached the plate and ate while the experimenter was close to the kennel were assigned to the “close” dogs (CD) group. This classification was later used as a factor in statistical analysis. Initially, the purpose of the test was to assess the dog’s behaviour in terms of their latency to approach an unfamiliar human when a feeding opportunity was close to the human. However, the preliminary analysis showed a clear dichotomous response of animals, observing that half of the dogs approached the plate located near the experimenter, whilst the other half did not approach. Hence, we decided to assess the behaviour response to the human as a dichotomous variable; 1) close: when the dog approached the plate with the experimenter nearby and 2) distant: when the dog was reluctant to approach and remained far away from the plate and the experimenter.

#### 2.2.2. Perception of Sucrose Solutions

Dogs were accustomed to sucrose solution tests by offering them two equidistant drinkers, during 20 min for four consecutive days (15:00 h). Solutions contained 2 L of water or 2 L of water plus 1% sucrose. The positions of solutions (left or right) were counterbalanced across animals and days. After this period, daily acceptability tests were carried out, in order to quantify the effect of sucrose concentration on animals’ solution intake (1%, 2%, 3%, and 4%) during 20 min for four consecutive days (one solution per day). Half the dogs were tested with the higher sucrose inclusion first and the other half with the lowest. Afterwards, two-choice preference tests were carried out (20 min) between the same sucrose solutions and water. Each comparison was tested in two consecutive days, where the position of sucrose and water solutions was counterbalanced across dogs and days. The consumption of solutions was estimated by the difference between the offered and remaining weight of each drinker and analysed according to animal HAT (CD or DD) in addition to other intrinsic variables of animals such as age and body weight.

#### 2.2.3. Perception of Monosodium Glutamate Solutions

The same dogs were accustomed to MSG solution tests by offering them two equidistant drinkers, during 20 min for four consecutive days (15:00 h). Solutions contained 2 L of water or 2 L of water plus 1 mM MSG solution. Additionally, inosine 5’monophosphate and guanosine 5’monophosphate (I + G) were incorporated at 2% of MSG inclusion as flavour enhancers. The positions of solutions (left or right) were counterbalanced across animals and days. After this period, daily acceptability tests were carried out in order to quantify the effect of MSG concentration (0, 0.1, 1, 10, 25, 50, and 75 mM) on animals’ solution intake during 20 min for seven consecutive days (one solution per day). Half the dogs were tested with the higher MSG inclusion first and the other half with the lowest. Afterwards, two-choice preference tests were carried out (20 min) between new dilute MSG solutions (2, 3, 4, and 6 mM) and water. We opted for the diluted concentrations as stated in the sucrose literature to estimate possible anhedonia symptoms and because the scarce information in domestic dogs against MSG intake. Each comparison was tested in two consecutive days where the position of MSG and water solutions was counterbalanced across dogs and days. The consumption of solutions was estimated by the difference between the offered and remaining weight of each drinker and analysed according to animal HAT (CD or DD) in addition to other intrinsic variables of animals such as age and body weight.

### 2.3. Statistical Analysis

Data collected during the acceptability and preference tests in each experiment were analysed by mixed linear models with the MIXED procedure of the statistical package SAS^®^ (SAS Inst. Inc., Cary, NC, USA). Sex and dogs body weight were included in explorative models. However neither in sucrose nor the MSG experiment did these variables affect the dog’s solution intake and so they were excluded. For the acceptability test, animals’ intake was analysed taking into account dog´s HAT (CD or DD), solution concentration, and their interaction. The age of dogs was considered as a covariate. For each preference test, animals’ intake was analysed through an ANOVA taking into account dog´s HAT (CD or DD), the consumed solution (sucrose/MSG or water), and their interaction. The age of dogs was considered as a covariate. The experimental unit (dog) was analysed as a repeated measure. Prior to ANOVA analysis, normality and homoscedasticity of the dataset were analysed by using the UNIVARIATE procedure with the Shapiro Wilk and O’Brien’s test, respectively. The mean values are presented as LSMeans considering a significance level of 5% adjusted by Tukey. A tendency was considered when 0.05 < *p* < 0.10.

## 3. Results

### 3.1. Sucrose Perception

**Figure 1:**
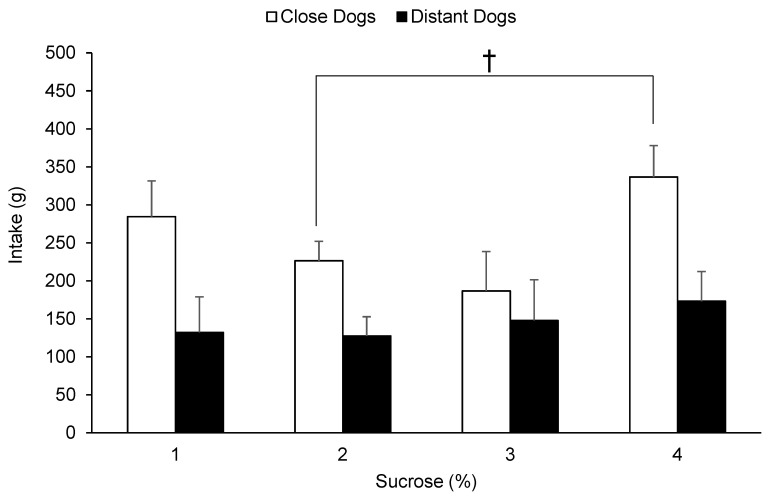
Consumption (least square means + standard error) of kennelled dogs in front of different sucrose solutions (1%, 2%, 3%, and 4%) during a 20 min acceptability test according to the human-approach test results (close or distant) previously assigned († *p* < 0.1).

Sucrose solution intake according to its concentration and animals HAT are presented in Figure 1. The acceptability of kennelled dogs was affected by sucrose concentration (F(3, 11) = 4.35, *p* = 0.029), observing higher intakes at the lowest and the highest sucrose inclusions. The HAT also had an effect on animals’ intake (F(1, 11) = 5.39, *p* = 0.041), where CD consumed more sucrose solutions than DG (259 vs. 145 g, EE 34 g) overall. Moreover, CD consumed more solution than DD at all concentrations offered, however, these differences did not reach significance. Dogs’ age and the interaction between HAT and concentration did not affect dogs’ intake (*p* > 0.05).

**Figure 2:**
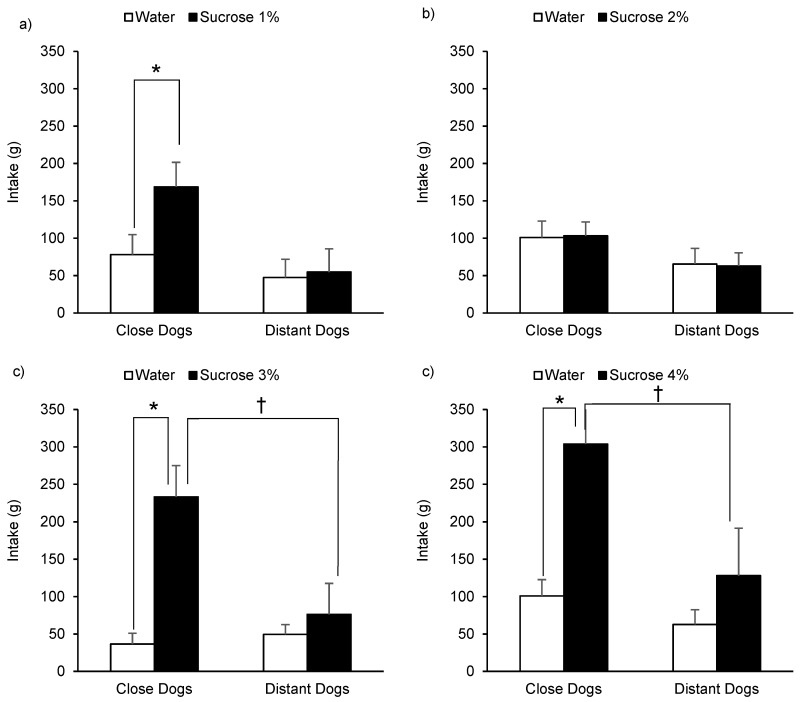
Consumption (least square means + standard error) of kennelled dogs in front of 1% (**a**), 2% (**b**), 3% (**c**), and 4% (**d**) sucrose and water solutions during a 20 min two-choice preference test according to the human-approach test results (close or distant) previously assigned (* *p* < 0.05; † *p* < 0.1).

Preference tests performed between each sucrose solution and water are presented in Figure 2. When dogs were tested to prefer between 1% sucrose solution and water (Figure 2a) they consumed more of the sweet solution (F(1, 11) = 5.45, *p* = 0.039). The intake during the test was affected by animals HAT (F(1, 11) = 4.67, *p* = 0.054), where CD consumed more than DD. Dogs intake tended to be affected by the interaction between the solution and HAT (F(1, 11) = 3.95, *p* = 0.072), where only CD significantly preferred the sucrose solution over water (*p* = 0.046). The age of animals did not have an effect on their consumption (F(1, 11) = 0.67, *p* = 0.432). When dogs were tested to prefer between 2% sucrose solution and water (Figure 2b), no effects of the solution (F(1, 11) = 0.00, *p* = 0.996), HAT (F(1, 11) = 3.09, *p* = 0.110), or the interaction between the solution and HAT (F(1, 11) = 0.02, *p* = 0.899) were observed. The age of animals tended to affect their consumption (F(1, 11) = 4.27, *p* = 0.063), however, no lineal relation was observed (*r* = 0.066; *p* = 0.736). When dogs were tested to prefer between 3% sucrose solution and water (Figure 2c) they consumed more of the sweet solution (F(1, 11) = 15.03, *p* = 0.003). The intake during the test tended to be affected by animals HAT (F(1, 11) = 4.29, *p* = 0.063) where CD consumed more than DD. Dogs intake was affected by the interaction between the solution and HAT (F(1, 11) = 8.70, *p =* 0.013), where only CD significantly preferred the sucrose solution over water (*p =* 0.003). The age of animals did not have an effect on their consumption (F(1, 11) = 0.76, *p =* 0.401). Finally, when dogs were tested to prefer between 4% sucrose solution and water (Figure 2d) they consumed more of the sweet solution (F(1, 11) = 8.25, *p =* 0.015). The intake during the test was affected by animals HAT (F(1, 11) = 9.59, *p =* 0.010) where CD consumed more than DD. Dogs intake was affected by the interaction between the solution and HAT (F(1, 11) = 5.92, *p =* 0.033), where only CD significantly preferred the sucrose solution over water (*p =* 0.015). The age of animals did not have an effect on their consumption (F(1, 11) = 2.55, *p =* 0.139). In both 3% and 4% sucrose vs. water tests, CD tended to consume more the sucrose solution than DD (*p* < 0.1).

### 3.2. Monosodium Glutamate Perception

**Figure 3:**
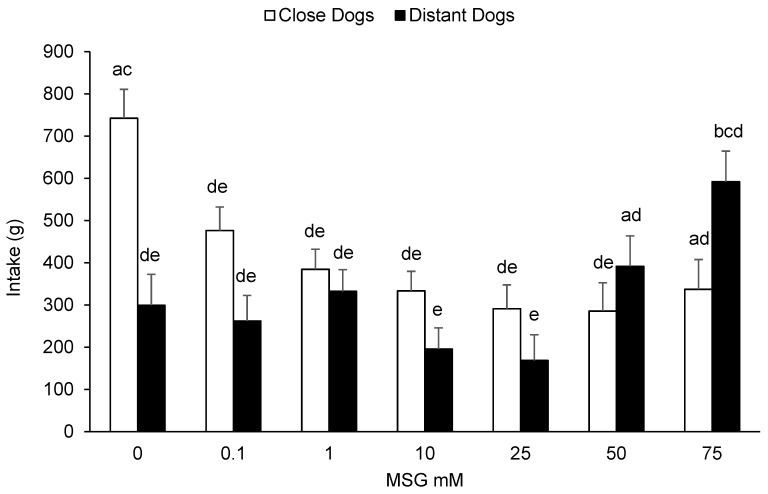
Acceptability of monosodium glutamate (MSG) solutions (least square means + standard error) at different dilute concentrations (0, 0.1, 1, 10, 25, 50, and 75 mM) in kennelled dogs during a 20 min test according to the human-approach test results (close or distant) previously assigned. Different letters (a, b, c, d, and e) represent statistical differences between columns (*p* < 0.05).

Dogs MSG solution intake according to the MSG concentration included and animals HAT are presented in Figure 3. The acceptability of kennelled dogs for MSG solutions was affected by its concentration (F(6, 12) = 40.65, *p* < 0.001), observing higher intakes at the lowest and highest MSG inclusions. HAT tended to affect animals intake (F(1, 12) = 4.01, *p =* 0.069), where CD consumed more MSG solutions than DD (407 vs. 320 g, EE 43 g) overall. The interaction between the solution and HAT also affected dogs consumption (F(6, 12) = 9.8, *p* < 0.001). As shown in Figure 3, CD presented higher intakes of MSG at the lowest concentrations whilst DD presented higher intakes of MSG at the highest concentrations. The age of animals tended to affect their consumption (F(1, 12) = 3.36, *p =* 0.092), however, no lineal relation was observed (*r* = −0.019; *p =* 0.8619).

**Figure 4:**
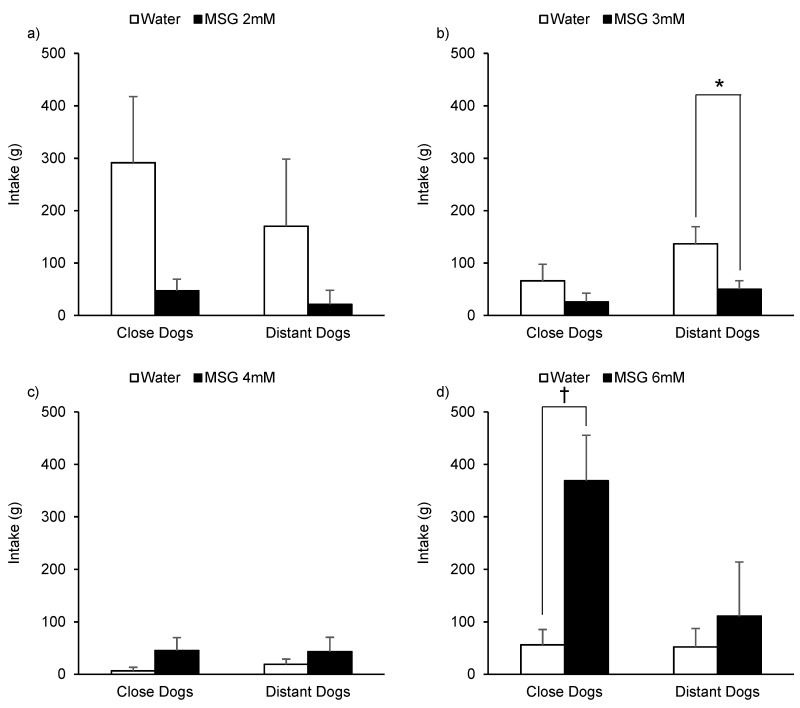
Consumption (least square means + standard error) of kennelled dogs in front of 2 mM (**a**), 3 mM (**b**), 4 mM (**c**), and 6 mM (**d**) monosodium glutamate (MSG) and water solutions during a 20 min preference test according to the human-approach test results (close or distant) previously assigned (* *p* < 0.05; † *p* < 0.1).

Preference tests between dilute MSG solutions and water are presented in Figure 4. When dogs were tested to prefer between 2 mM MSG solution and water (Figure 4a) they tended to consume more of the water solution (F(1, 8) = 4.17, *p =* 0.076). No effects of the HAT (F(1, 8) = 0.69, *p =* 0.430), the interaction between the solution and HAT (F(1, 8) = 0.25, *p =* 0.633), or the age of animals (F(1, 8) = 3.18, *p =* 0.122) were observed. When dogs were tested to prefer between 3 mM MSG solution and water (Figure 4b) they consumed more water (F(1, 9) = 27.54, *p* < 0.001). The intake during the test was not affected by animals HAT (F(1, 9) = 1.92, *p =* 0.199). The interaction between HAT and the solution consumed tended to affect dogs intake (F(1, 9) = 3.75, *p =* 0.085) where DD but not CD significantly preferred the water over the MSG solution (*p =* 0.004). The age of the animals did not affect solution intake (F(1, 9) = 0.36, *p =* 0.564). When dogs were tested to prefer between 4 mM MSG solution and water (Figure 4c) the only variable that affected their consumption was their age (F(1, 8) = 9.13, *p* < 0.017) where older dogs drunk more. The solution (F(1, 8) = 2.33, *p* < 0.165), HAT (F(1, 8) = 0.07, *p* < 0.794), and their interaction (F(1, 8) = 0.12, *p =* 0.734) did not present a significant effect on dogs consumption. Finally, when dogs were tested to prefer between 6 mM MSG solution and water (Figure 4d) they tended to consume more of the umami solution (F(1, 8) = 4.96, *p =* 0.057). The intake during the test was affected by animals HAT (F(1, 8) = 5.05, *p =* 0.055) where CD consumed more than DD (213 vs. 81 g, EE 58 g). The interaction between the solution and HAT did not have an effect on dogs’ intake (F(1, 8) = 2.32, *p =* 0.167), however, only the CD tended to exhibit a higher consumption of the MSG solution (*p =* 0.076). The age of animals did not affect their intake (F(1, 8) = 0.52, *p =* 0.491).

## 4. Discussion

The present study evaluated the acceptability and preference between dilute sucrose or monosodium glutamate solutions with respect to drinking water in kennelled dogs, in order to determine the possible presentation of anhedonia in this population according to the dog’s strategies to cope with stressors. The dogs´ response to a human-approach test with a feeding opportunity was previously assessed as a measure to describe the coping style of the animals, and thus differences in strategies to face chronic stress, which could be reflected as anhedonia and feeding behaviour changes. It was observed that DD (reactive) exhibited a lower intake of both sucrose and MSG dilute solutions compared with CD (proactive). However, an interesting interaction between HAT and MGS concentration over dogs’ consumption was observed, where DD exhibited higher consumption of MSG at the highest concentrations than CD, supporting the fact that the intake of chronically stressed animals depends on how palatable is the food or solution source [48,49]. In addition, DD did not prefer sucrose or MSG solutions over water at any dilute solutions offered. Taken together, these results suggest that dogs that are categorized as reactive animals under a kennel environment could see diminished their ability to perceive palatable sources, thus reflecting anhedonia (i.e., a reduction in the experience of pleasure).

### 4.1. Coping Styles and Anhedonia

Dogs that are kennelled for a long time usually live under suboptimal conditions due to a variety of psychogenic stressors, including spatial, feeding, and social restriction, added to the lack of control and environment complexity [50]. These dogs can remain in kennels for several months and in some cases years, such as the ones used in these experiments, which were maintained for pet food industry feeding trials. If they fail to adapt to adverse challenges typical of this environment, they are at risk of becoming chronically stressed [50]. In our study, dogs were on average 5 years under these conditions, where they were: a) spatially restrained; b) socially isolated and kept for more than 20 h per day without the possibility of tactile or visual interactions with conspecifics or humans; c) limited in their feeding frequency, being fed once a day; and d) housed in a sensory deprived environment without any type of environmental enrichment. Although in the present study no indicators were used to evaluate stress or welfare, due to their kennelling managements previously described, they were considered to be at least at risk of suffering chronic stress, according to previous studies performed in kennelled dogs in similar environments [23,51,52], which could trigger anhedonia in certain individuals.

In the present study, dogs showed different responses when they were exposed to a food source with an unfamiliar person nearby (human-approach test with a feeding opportunity). This test resembles in some respect to human-approach tests commonly used to describe the coping strategies in domestic animals [41,43,47,53], including dogs [45,46]. Generally, dogs tend to exhibit two types of behavioural response when they are confronted with an unknown human. Some dogs behave in an impulsive manner, approach directly towards the human, while others tend to avoid any type of interaction reacting with submission [46]. Such behaviours have been related to different coping strategies (i.e., proactive and reactive) both in dogs and other domestic animals. In the present study, DD showed a reactive (passive) coping strategy because they were reluctant to approach the food source located near the person. CD were more active, approaching the food source with the person nearby. Thus, these dogs could be labelled as displaying a proactive coping strategy [46]. These findings are in agreement with previous studies, where individuals classified as proactive approached the stimulus, exhibiting shorter latencies to explore novel stimuli, less latency to feeding and a greater locomotive activity [24,54,55], whilst individuals classified as reactive display immobility behaviour, typically associated with a passive response [24,56]. Despite the fact that coping styles are stable and consistent over time and across situations (social and non-social), other tests to assess coping styles, such as a novel object test or an open-field test would have been beneficial to include to further corroborate the coping styles found in the present study.

The different responses obtained regarding sucrose and MSG acceptability and preference over water between animals could be attributed to individual stress sensitivity differences and coping styles [36]. Proactive animals tend to be quicker to explore a novel environment and have higher locomotive activity than reactive individuals. In addition, proactive individuals try to manipulate the situation, exhibit higher levels of dopamine than reactive animals, show behavioural patterns that are largely independent of external stimuli, and tend to form routines [25,36,57]. It has been proposed that these characteristics predispose proactive animals to develop stereotypic behaviours as a strategy to cope with chronic stress. Therefore, future experiments could examine differences in the presentation of abnormal behaviours between reactive and proactive kennelled dogs. This could be of great significance to more accurately distinguish between coping strategies observed and how they cope with chronic stress. On the other hand, reactive animals are slower in taking decisions, react with immobility, and they tend to adjust to a situation in a more passive way rather than manipulating it [25,58], showing lower basal dopamine levels [36]. In addition, reactive individuals faced with chronic stressors are more prone to experience depression [35,36,37]. Both stereotypy and depression serve to dearouse the animal and minimize the effects of stress, but while the appetitive loop in proactive animals will continue until the individual is aroused enough to redirect to stereotypy, in reactive individuals depression will act negatively on motivation, appetitive behaviours, and pleasure perception, therefore showing anhedonia [36], as is the case for the DD animals.

On the other hand, it is also relevant to highlight that since proactive animals are more prone to form routines, they could adapt more easily within stable and predictable environment conditions, such as the kennel environment described in the present study. Social stress studies show that proactive individuals are resilient under stable environmental conditions but vulnerable when outcome expectancies are violated [24]. In a study done by De Palma et al. [59], dogs that showed a confident-independent temperament in a familiar context (within the shelter) did not show willingness to approach a novel situation and to take risks in an unknown context (the street outside the shelter). On the other hand, reactive animals are more cautious and sensitive to external stimuli, which they analyse while trying to adjust to the situation, and are rather flexible and seem to adapt more easily to a changing environment [24,59,60]. Therefore, reactive dogs under a kennel environment (typically stable) could be less efficient with their coping strategy under this context, being stress sensitive reactive dogs more prone to experiencing anhedonia. However, the results obtained must be interpreted with caution, since our study did not evaluate stress or welfare in these animals. Accordingly, future studies could examine physiological and/or behavioural changes between different coping styles in kennelled dogs, in order to determine which individuals exhibit signs of chronic stress or poor welfare, and compare different responses between them in relation to their coping styles.

### 4.2. Sucrose Perception

The sweet taste in most mammals predicts the energy that a food contains, being an accepted and preferred flavour due to the reward of its consumption [61,62,63]. Although the dog maintains strong preferences for protein foods [64], domestication has created adaptive physiological changes that have generated preferences for simple carbohydrates [65], allowing them to consume and digest certain amounts of sugars, such as sucrose [38]. In the present experiment dogs showed adequate acceptability for the sucrose solutions offered where the most consumed concentrations were 1% and 4%, which could indicate a search for the energy resource and greater palatability respectively. The DD showed lower acceptability than CD when dilute sucrose was offered, which could indicate depressive-like behaviours that do not allow them to perceive the energy reward as anhedonia research propose [5,6,8]. Probably these DD may need more sucrose to exhibit the same amount of intake than CD as experiments in other species have previously described where chronic stress promotes palatable feeding [48,49], which reduces signs of stress. Since in the present experiment sucrose was included only until 4%, future research will need to explore if the bidirectional effect is observed in reactive animals when higher amounts of sugar is included.

Although the dogs were able to prefer the sweet solution over water in most cases, the interaction between the solution (sucrose or water) and dogs’ approach to the human indicates that only the CD preferred the sweet solutions. If the results obtained from the human-approach test with a feeding opportunity were therefore related to the coping styles of these animals, the results found would go in the expected direction. CD would be able to perceive the hedonic value of sucrose preferring it over water, showing higher consumptions than DD, which if suffering chronic stress due to their long-term housing and husbandry conditions, might react with depression-like behaviours [36], manifesting failures in the perception of palatable sources such as sucrose, hindering their preference over water and showing lower consumptions of these diluted solutions as was described in other species that suffer anhedonia [8,9].

### 4.3. MSG Perception

To our knowledge, this is the first study that uses MSG to evaluate anhedonia in domestic dogs using preference tests. Previous studies used this compound for other purposes, such as assessing intestinal motility [66] or recording the activity of the tympani chorda nerve [67]. When evaluating the basal consumption of MSG solutions using acceptability tests, it gradually decreased as the inclusion doses were increased. However, at 50 and 75 mM, MSG intake increased considerably. The increase in dogs MSG consumption at the highest doses could be due to greater hedonic reactions, as they are associated with a greater reward, as occurs in other animals such as pigs [68]. However, the consumption curve is different from that observed in other omnivorous species such as rats [69] and pigs [68], where in both species the consumption increases until reaching a point in which it remains constant. Additionally, the consumption of MSG does not agree with consumption of other palatable solutions such as sucrose, where when increasing the level of inclusion its consumption increases until reaching a plateau after which consumption decreases gradually in species such as pigs [68] and rats [70]. Due to the scarce information in domestic dogs against MSG intake, it was difficult to estimate the appropriate doses to use in preference tests opting for the diluted concentrations as stated in the sucrose literature to estimate possible anhedonia symptoms.

Although there was not a clear effect of coping style on MSG acceptability, an interaction between the dog response to the human approach and offered concentrations was found. At intermediate doses of MSG, CD had higher MSG consumption than DD, a situation that was reversed by presenting higher doses of MSG. This could again reflect different coping strategies of animals in response to stressful situations (proactive or reactive individuals), which can be determined by genetic factors, previous experiences, and the environment [25,56]. In dogs, these different coping styles were evaluated in stressful situations, determining that proactive individuals manifested low magnitude changes in their cortisol levels (16.5% on average). However in reactive individuals there is a greater change in their cortisol levels, reaching variations of up to 80% [46]. Therefore, if the reaction evaluated in the dogs in the present study reflected their possible "coping strategies", the DD could react differentially under chronic stress due to the kennelling conditions, showing depressive-like behaviours, reflecting anhedonia. This reduces the consumption of palatable umami solutions at low concentrations, but when its concentration increases, anhedonia promotes the intake of highly rewarding solutions [48,49,71].

When the dogs were exposed to preference tests between MSG and water, no interesting results were observed until we reached 6 mM where, similarly to sucrose vs. water comparisons, only CD tended to prefer the umami solution. This could indicate that similar to dilute sucrose, MSG could be a valid solution to explore anhedonia through preference tests due to its hedonic value for this species [39]. However, more research is needed to elucidate, which would be the appropriate concentrations to describe anhedonia.

## 5. Conclusions

Kennelled dogs are exposed to several stressors, and if they fail to adapt, they are at risk of becoming chronically stressed, to which dogs can react with different individuals’ coping styles. Under these conditions, different coping strategies can alter their hedonic preferences, reflected in differences in their sucrose and MSG consumption and preference over water. To our knowledge, this is the first study that assessed anhedonia in domestic dogs subjected long-term to a kennel environment. In this study, dogs kept in a stable and predictable environment responded differently depending on their coping style, where reactive dogs would be more prone to exhibit anhedonia, which is considered as a depressive-like behaviour. Further studies are needed to examine physiological and/or behavioural changes in kennelled dogs, in order to determine which individuals exhibit signs of chronic stress or poor welfare, and compare different responses between them in relation to their coping styles.
